# Automated AI detection of thoracic aortic dissection on CT imaging

**DOI:** 10.1186/s41747-025-00640-8

**Published:** 2025-10-22

**Authors:** Tobias Norajitra, Michael A. Baumgartner, Lucas R. Cusumano, Jesus G. Ulloa, Christian S. Rizzo, Florian Haag, Alexander Hertel, Nils A. Rathmann, Steffen J. Diehl, Stefan O. Schoenberg, Klaus H. Maier-Hein, Johann S. Rink

**Affiliations:** 1 Division of Medical Image Computing, German Cancer Research Center (DKFZ), Heidelberg, Germany; 2 Pattern Analysis and Learning Group, University Hospital Heidelberg, Heidelberg, Germany; 3 Department of Radiology, Ronald Reagan UCLA Medical Center, Los Angeles, CA USA; 4 Division of Vascular and Endovascular Surgery, David Geffen School of Medicine at UCLA, Ronald Reagan Medical Center, University of California Los Angeles, Los Angeles, CA USA; 5 Department of Radiology, Hospital Alvarez, Ciudad Autonoma de Buenos Aires, Buenos Aires, Argentina; 6 Department of Radiology and Nuclear Medicine, University Medical Centre Mannheim, Mannheim, Germany

**Keywords:** Aortic dissection, Artificial intelligence, Computed tomography angiography, Deep learning, Tomography (x-ray computed)

## Abstract

**Background:**

Aortic dissection (AD) is a life-threatening condition. We developed an artificial intelligence (AI) algorithm capable of robust, accurate, and automated AD detection and sub-classification.

**Materials and methods:**

Based on 2010–2023 data from Mannheim University Medical Centre, heterogeneous internal training cases with confirmed AD (*n* = 70) were manually segmented and, together with non-AD cases (*n* = 87), used for training of a convolutional neural network (CNN; U-Net architecture) configured using the nnU-Net framework. Internal test dataset was composed of 106 cases. The external test was performed on a public dataset: 100 AD cases from ImageTBAD, Guangdong Provincial People’s Hospital, China, and 38 non-AD cases from the AVT dataset (multiple sources). Model performance was evaluated by area under the receiver operating characteristic curve (AUROC), area under the precision-recall curve (AUPRC), sensitivity, specificity, precision, and F1-score, and by investigating performance on different subsets of cases. Confidence intervals were determined using DeLong’s method and bootstrapping.

**Results:**

The best-performing algorithm achieved an AUROC of 98.7% (95% CI: 96.1–100.0%) and an AUPRC of 98.9% (96.0–100.0%) on the internal test dataset, 97.0% (94.7–99.3%) and 99.06% (98.0–99.7%) on the external test datasets, respectively. In the internal test dataset, of 15 unsuspected AD cases, 14 (93.3%) were successfully detected by the algorithm. On the external test dataset, sensitivity, specificity, precision, and F1-score were 92.0%, 100.0%, 100.0%, and 95.8%, respectively.

**Conclusion:**

The developed AI pipeline highlighted the capability of optimized CNNs to reliably detect AD across heterogeneous multicenter datasets. The resulting tool will be made publicly available for further scientific evaluation.

**Relevance statement:**

Artificial Intelligence demonstrated promising potential to detect AD on heterogeneous thoracic CT imaging data.

**Key Points:**

- Early detection of aortic dissection (AD) is crucial for timely treatment.
- A modern convolutional neural network (CNN) achieved 93.5% sensitivity and 100.0% specificity for AD detection on multicenter, heterogeneous CT data.
- These results demonstrate the potential of streamlined, optimized CNNs for robust AD detection on CT, supporting fast clinical response.

**Graphical Abstract:**

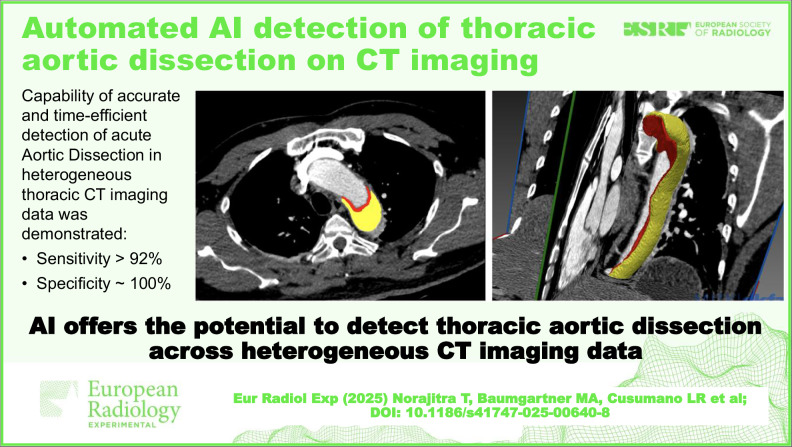

## Background

Acute aortic syndrome encompasses the life-threatening diseases of aortic dissection (AD), intramural hematoma and penetrating aortic ulcer [1]. AD is characterized by acute tearing of the aortic intima, causing blood inflow into the aortic wall. AD represents a significant healthcare burden with a reported 2.6 to 7.2 cases per 100,000 person-years in Western countries and an in-hospital mortality of 30.1% in women and 21.0% in men [2].

Modern AD management relies on early diagnosis to enable appropriate therapy. Type A dissections originate in the ascending aorta and constitute a surgical emergency; type B dissections may be managed by medication and elective surgery [3]. The earliest possible diagnosis is crucial to initiate goal-directed management, as mortality typically peaks within the first 48 h with nearly half of patients dying within 7 days, mainly secondary to aortic rupture, cardiac tamponade or visceral ischemia [4]. Electrocardiographically gated computed tomography angiography (CTA) has evolved as the diagnostic standard in this setting [5, 6]. However, nonspecific symptoms may delay diagnosis [6, 7]. Moreover, image misinterpretation can delay treatment [8], particularly during emergency conditions when no specialized cardiovascular radiologists are available.

Artificial intelligence (AI) has shown promise in clinical triage, potentially reducing delays in urgent cases [9]. AD detection represents a potentially valuable but challenging use case given the variable morphology, limited number of training cases and non-standardized imaging protocols arising from cases without prior AD suspicion. As previously proposed solutions have in many cases been developed and tested with highly preselected imaging data, modern CNN-based approaches could offer the potential to deal with highly heterogeneous data and improve generalizability. Moreover, efficient AI-based detection should aim to optimize both sensitivity and specificity while providing substantial automation and robustness.

The aim of this work was to test the feasibility of developing a robust automated AI algorithm for detecting acute AD in heterogeneous emergency thoracic CT scans. Performance was to be validated in detail on additional heterogeneous, internal and external data collections arising from routine clinical imaging.

## Materials and methods

This study was approved by the institutional ethics committee (see Declarations). Written informed consent was not necessary due to retrospective data collection. Adherence to internationally accepted standards in machine learning studies was documented by the CLAIM checklist (Supplementary Table S5).

### Data collection

**Fig. 1:**
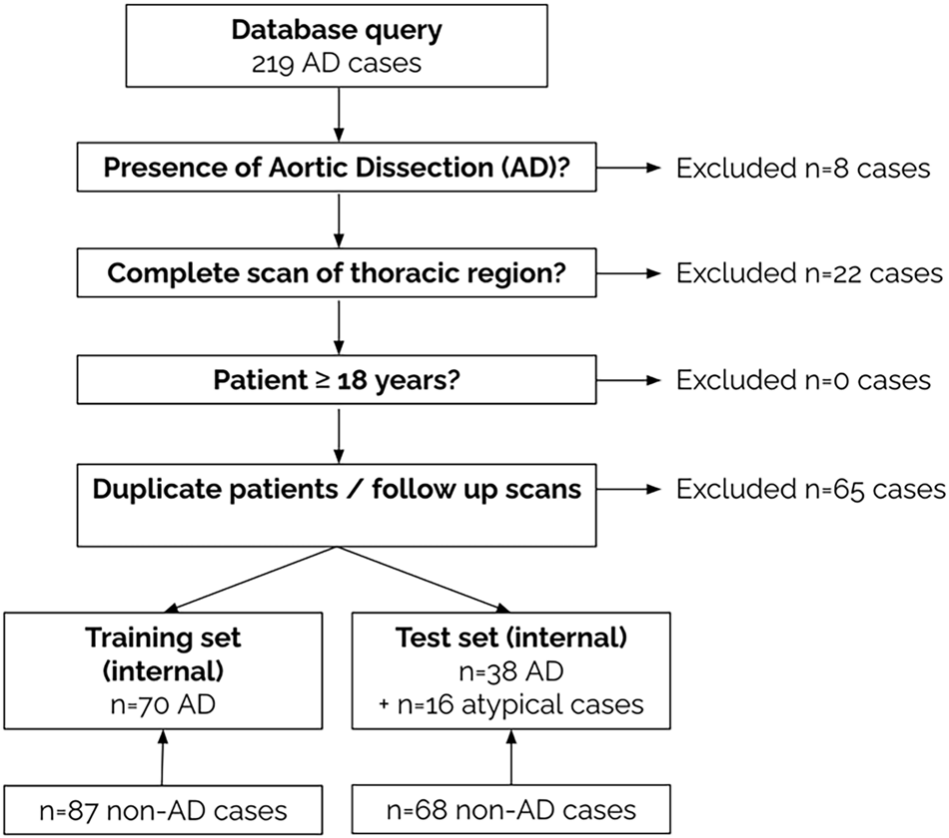
Data collection flowsheet. Overview of the data collection process for the internal training and test datasets from the clinical systems. In most cases, patient datasets were to be excluded because incomplete imaging data were present or because it was follow-up imaging of a known aortic dissection

**Fig. 2:**
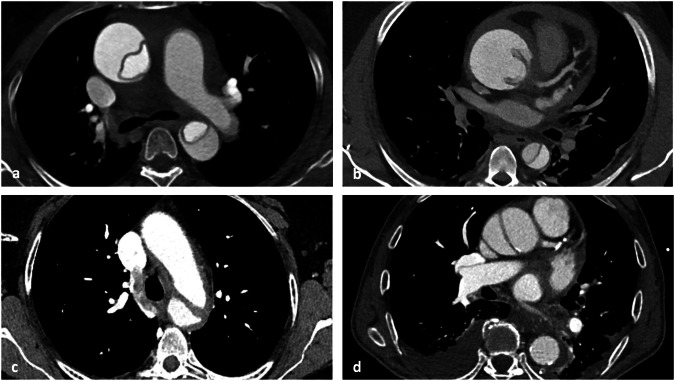
Exemplary training cases with acute AD. Cases of acute AD on contrast-enhanced axial images from the training dataset. **a**, **b** Type A dissection affecting the ascending and descending aorta and partly expressing hemopericardium. **c** Type B dissection of the descending aorta. **d** Type A dissection not extending into the descending aorta. Anatomy of the aorta and AD extent, as well as the presence of side-branch dissection, contrast phases and image quality varied strongly between cases. AD, Aortic dissection

CT studies of imaging-confirmed acute AD originating from Mannheim University Medical Centre were retrospectively identified in the Radiology Information System from January 01, 2010, to February 28, 2023. Cases were included if CT covered the thoracic region, regardless of contrast phase (CTA, Pulmonary Artery contrast or mixed phase) or electrocardiographic gating. Contrast was achieved using iodine-based contrast agents, in the vast majority of cases utilizing Imeron 350 (Bracco Imaging Deutschland). Of the AD patients presenting with visible abdominal AD, 40 had been part of a previous study focusing solely on the abdominal regions [10]. This study was the first to analyze the collected thoracic imaging data. Patients < 18 years or with pre-existing AD were excluded (Fig. 1). Imaging data of patients without AD in contrast phases comparable were randomly collected and included. Patient age and sex were recorded from DICOM data. Where available, pre-imaging suspicion for AD was analyzed from the imaging request text in the DICOM data, and these cases were then separated. Patient identifying information was entirely removed, and annotations were stored in an Excel spreadsheet (Microsoft Corporation). Exemplary images are shown in Fig. 2.

Algorithm testing was performed first on a separated internal dataset (random 40% test split of all internal cases, remaining 60% used for training). Thoracic ADs of atypical anatomy, such as subtle dissection membranes with no visible false lumen (Supplementary Fig. 1), were additionally collected in another internal dataset (*Atypical cases (Test)*) and remained excluded from training. For the external testing, publicly available CTs were collected from the ImageTBAD dataset (from Guangdong Provincial Peoples’ Hospital) for AD cases [11], and AVT datasets for non-AD cases [12] described to contain cases from two hospitals in the US and one hospital in China (details in Supplementary Table S1).

### Training data annotation

Images of AD patients were manually segmented using MITK v2022.10 (German Cancer Research Center) by a board-certified radiologist (J.S.R., 5 years of experience in cardiovascular imaging). The images were independently read by a board-certified interventional radiologist (N.A.R., > 10 years of experience). Disagreements were decided by a board-certified senior interventional radiologist (S.J.D., > 20 years of experience). If present, entire false lumen of ascending AD (label 1), dissection membrane (label 2), false lumen of descending AD (label 3), hemopericardium (label 4), aortic wall hematoma (label 5), false lumen in brachiocephalic trunk (label 6), carotid artery right (label 7), subclavian artery right (label 8), carotid artery left (label 9) and subclavian artery left (label 10) were segmented.

### Neural network training

Image-based AD detection can be approached, for instance, via object detection, instance segmentation, semantic segmentation, or image classification. We opted for a strong and well-generalized supervised AI approach to perform semantic segmentation. To account for individual morphologic heterogeneity of AD, all retrospectively available CT scans were to be included, not limited to CTA imaging, but also to include other contrast phases. We trained models to segment false aortic lumina and dislocated membrane as main indicators for dissections. Optionally, we considered indirect signs of AD (supra-aortic branches, aortic wall hematoma, hemopericardium). We relied on nnU-Net [13] as a state-of-the-art segmentation framework to optimally train deep segmentation models and perform predictions. By formulating AD detection as a semantic segmentation task, we avoided additional methodological complexity while leveraging the strong generalizability and capacity of the commonly used U-Net architecture. Specifically, nnU-Net achieves generalizability through extensive data augmentation—rotation, scaling, elastic deformation and gamma correction during training, mirroring during training and segmentation—and through model ensembling over 5-fold cross-validation splits. Hierarchical feature representations are learned by the neural networks, enabling both contextual and high-resolution segmentation of target structures. During training, a combined Dice and cross-entropy loss was minimized using the Adam optimizer with an initial learning rate of $3\times {10}^{-4}$, reducing the learning rate by a factor of 5 for stagnating loss during 30 epochs.

We trained nnU-Net V1 without additional modifications on expert-annotated CTA data for subjects with and without AD. We compared incorporating only AD cases *versus* also adding non-AD training cases. CT images were preprocessed by nnU-Net, *i.e*., by clipping intensities to the [0.5, 99.5] percentile range of masked intensities in the training data, followed by z-score normalization. During inference, nnU-Net produced an ensemble segmentation for each image after averaging the softmax probabilities across the trained fold models.

### Labeling configurations

**Fig. 3:**
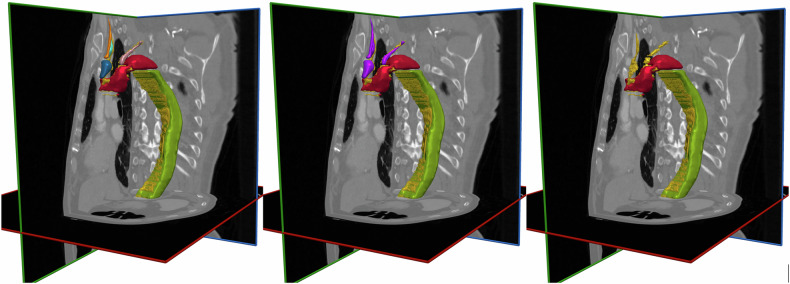
Labeling configurations for training data. AD detection performance was evaluated using three different input label configurations for segmentation model training. Labels for the false lumen of ascending AD (label 1: red), false lumen of descending AD (label 2: green), and the dissection membrane (label 3: yellow) were identical in all configurations. Configuration A (**left**): Included additional separate labels for hemopericardium (label 4), aortic wall hematoma (label 5), false lumen in brachiocephalic trunk (label 6: blue), carotid artery right (label 7: orange), subclavian artery right (label 8: green), carotid artery left (label 9: yellow-orange), and subclavian artery left (label 10). Configuration B (**middle**): combined labels 4–10 into a single additional label (purple). Configuration C (**right**): only labels 1, 2, and 3 used during training. AD, Aortic dissection

Based on separate labeling of 10 different AD-related features (Fig. 3), three possible label groupings were compared: (A) separate labels for each of the 10 features; (B) separate labels for false ascending and descending AD lumina and dissection membrane, jointly labeled hemopericardium and aortic wall hematoma, and jointly labeled dissection of supra-aortic branches (5 labels); and (C) separate labels for false ascending and descending AD lumina and dissection membrane (3 labels).

Labeling ambiguity can arise when adjacent anatomical regions overlap (*e.g*., false AD lumen and membrane), but each image voxel can only be assigned a single label. In such cases, occlusion of the thin membrane (1 or 2 voxels in-plane) by adjacent AD lumina can occur if the latter are prioritized during voxel-to-label assignment, thus aggravating label class imbalance. Therefore, we tested two additional label assignments, prioritizing either membrane or false AD lumina.

### Classifier decision-making

We aggregated automatic segmentation labels to arrive at a robust AD detection. Specifically, we considered AD present if at least 2 out of 3 segmented regions of interest (ascending & descending false AD lumina & membrane) exceeded a specified volume threshold. An optimal threshold was derived as part of the receiver operating characteristic analysis for the AD detector. We defined an unbounded classifier decision function ${f}({X}_{i})$ representing confidence of patient $i$ exhibiting AD. The decision function was calculated as the median of the three segmented ROI volumes in image ${X}_{i}$:

$$\,f({X}_{i})={median}({V}_{i,{ascending}},\,{V}_{i,{descending}},\,{V}_{i,{membrane}})$$

*i.e*., for segmented ascending and descending false AD lumina and membrane. Thus, samples are classified as AD if at least two segmented volumes exceed the decision thresholds set during receiver operating characteristic analysis. Optimal threshold ${\theta }_{{opt}}$ was derived at maximum value for Youden’s Index ${J}_{{\theta }_{{opt}}}$:

$${J}_{\theta }\,=\,{{sensitivity}}_{\theta }\,+\,{{specificity}}_{\theta }\,-\,1$$

$${\theta }_{{opt}}={argmax} \, {J}_{\theta }$$

with ${{sensitivity}}_{\theta }$ and ${{specificity}}_{\theta }$ based on classifier prediction

$${{pred}}_{i,{\rm{\theta }}}=\left\{\begin{array}{ll}P, & f({X}_{i})\ge {\rm{\theta }}\\ N, & {else}\hfill \end{array}\right.$$

for subject $i$ and decision threshold $\theta$ (P = predicted AD, N predicted non-AD). In favor of increased sensitivity, we adjusted the decision threshold to a slightly lower ${\theta^{\prime} }_{{opt}}$.

We additionally performed the detection of Stanford type A ADs. Only in cases marked as dissections, we predicted type A, if segmented ascending false lumen surpassed the given threshold.

Segmentations were carried out at test time either on an Nvidia TitanXP or Nvidia Tesla V100.

### Algorithm performance measurement and statistical analysis

Detection performance on different datasets was measured as the area under the receiver operating characteristic curve (AUROC, %). Sensitivity, specificity, and Cohen κ for AD detection were calculated for classifiers at ${\theta^{\prime} }_{{opt}}$ near maximum Youden’s index. Confidence intervals and *p*-values were determined using DeLong’s method (RStudio v2023.06.2, R version 4.3.1, pROC package v1.18.5). Differences were considered statistically significant for *p* < 0.05. In addition, detection performance was evaluated in terms of the area under the precision-recall curve (AUPRC, %). For this evaluation, precision and recall were determined at the operating point corresponding to the maximum F1-score of the detector. Confidence intervals (CIs) were computed via bootstrapping (RStudio v2023.06.2, R version 4.3.1, PRROC package v1.4). The AD-positive and AD-negative cases from both the internal test set and the atypical cases dataset were additionally read by two advanced-level radiology residents who typically cover emergency imaging shifts. Cases were presented in random order. The first reading was conducted in the Emergency Department with distractions present, whereas the second reading was conducted in a controlled, quiet research setting. Imaging request reasons were presented along with the images. Sensitivity, specificity and precision for AD detection and type A detection were subsequently calculated. These results were compared to the algorithm’s performance.

## Results

### Patient characteristics and AD morphology

Of the retrieved 219 potential AD cases, 95 were excluded (Fig. 1). The internal dataset was split into a training dataset (*n* = 157 cases) and a test set (*n* = 106 cases). Of all collected internal AD cases, in 96 cases, complete imaging request texts were available. In 45 of these cases (47.4%), there was no suspicion of AD before CT imaging. In the training set, 32.5% of cases (51/157) were females; in the test set, 38.7% of cases were females (41/106). In the training set, age was 62.6 ± 16.9 years (mean ± standard deviation; range 18–93); in the test set, age was 65.3 ± 14.6 years (24–93). Among the AD cases, 88.6% (62/70, training set) and 44.7% (17/38, test set) presented with dissection of the ascending aorta, 65.7% (46/70) (training set) and 100% (38/38, test set) with dissection of the descending aorta. Hemopericardium was present in 2.9% (16/70, training set) and in 26.3% (10/38, test set). Dissection of the aortic arch branches was present in 50.0% (35/70, training set) and in 18.4% (7/38, test set) (Table 1).

**Table 1:** Number of included cases in the internal and external datasets, as well as basic patient characteristics

	Internal set			External
	Training	Test	Atypical cases (Test)	Test
Cases/patients	157 (70 AD)	106 (38 AD)	16 AD cases	138 (100 AD)
% female	32.5% (51/157)	38.7% (41/106)	50.0% (8/16)	31% (31/100) of AD cases, control cases unknown
Stanford A %	88.6% (62/70)	44.7% (17/38)	87.5% (14/16)	0% (0/100)
Patient age	62.6 ± 16.9 years (18–93)	65.3 ± 14.6 years (24–93)	66.50 ± 16.68 years (21–92)	52.5 ± 11.3 years for AD cases, others unknown
CT hardware	Siemens SOMATOM Emotion 16 (slices), Sensation (64 slices) Definition Flash (256 slices), Force (384 slices), AS+ (128 slices)			AD cases: Siemens (23%) and Philips (77%) scanners; control cases: various scanners.
Slice thickness	1.98 ± 1.35 (1–6) mm		1.58 ± 1.15 (0.6–5.0) mm	1.64 ± 1.22 (0.5–5) mm
CT Protocol and contrast (CTA without ECG gating/CTA + gating/Trauma protocol/Pulmonary Embolism protocol)	37.8%/33.6%/9.5%/19.1%		18.8%/56.3%/25.9%/0.0%	100% CTA, ECG gating unknown

Whereas the internal data was entirely derived from different Siemens CT scanners, the external test set relied on data from different CT vendors. In the internal cases, different CT protocols with respect to contrast phase were utilized, whereas the external set only contained CTA scans

*AD* Aortic dissection, *CT* Computed tomography, *CTA* Computed tomography angiography, *ECG* Electrocardiogram

### Training and validation results

Label configuration B with grouped labels, prioritized membrane, and including healthy cases produced overall best results and was selected as the primary model.

An AUROC (%) of 97.0 (95% CI: 94.3–99.8) was achieved during 5-fold cross-validation on the training set, 98.7 (95% CI: 96.1–100.0) on the internal test set, 93.75 (95% CI: 85.4–100.0) on the internal atypical ADs and 97.0 (95% CI: 94.7–99.3) on the external test set. Similarly, AUPRC (%) of 97.6 (95% CI: 94.9–99.4), 98.9 (95% CI: 96.0–100.0), 91.9 (95% CI: 78.7–100.00), and 99.1 (95% CI: 98.0–99.7) were achieved on these datasets.

**Fig. 4:**
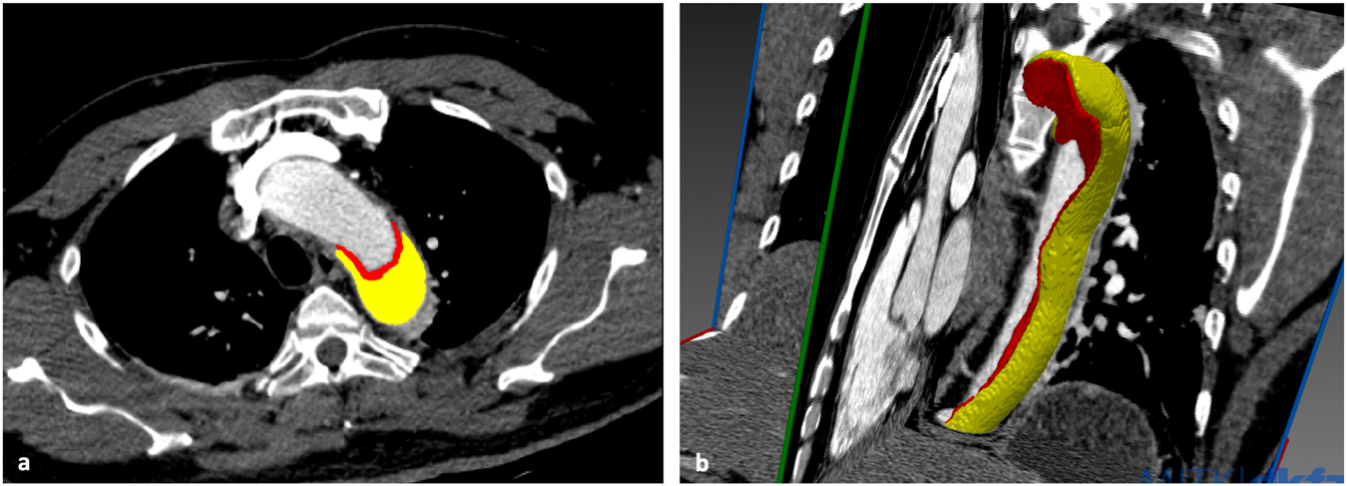
Automatic segmentation results. Example of segmentation capabilities of the proposed algorithm in a patient with type B aortic dissection (**a** two-dimensional axial view; **b** three-dimensional view). Yellow areas represent the resulting automated segmentation of the false lumen in the descending aorta; red areas represent the segmented dissection membrane

**Fig. 5:**
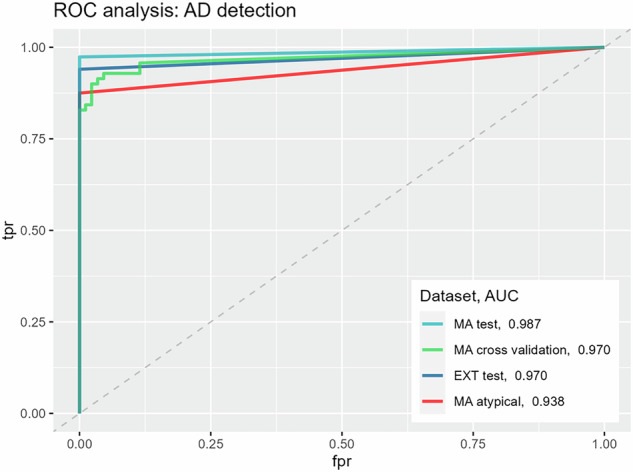
Algorithm performance on different datasets. The selected best version of the proposed method (grouped labels, included AD-negative cases) achieved high AUROC scores for AD detection across various datasets: 97.0% (95% confidence interval 94.3–99.8) for the internal training dataset (MA cross-validation, green), 98.7% (96.1–100.0) on the internal test set (MA test, cyan), and 97.0% (94.7–99.3) in the external test set (EXT test, dark blue). Rare, atypical cases were more difficult to detect automatically (AUROC: 93.8%, 85.4–100.0) (MA atypical, red). AD, Aortic dissection, AUROC, Area under the receiver operating characteristic curve, EXT, External, MA, Mannheim University Hospital (internal cases)

An AD detector selected at optimal decision threshold ${\theta^{\prime} }_{{opt}}$ as determined on the internal training set, achieved sensitivities of 95.7% (train, 5-fold cross-validation, 67/70), 97.4% (test internal, 37/38), 92.0% (test external, 92/100) and 81.3% (atypical cases, 13/16), and specificities of 88.5% (train, 5-fold cross-validation, 77/87), 100.0% (test internal, 68/68), 100.0% (test external, 38/38) and 100.0% (atypical cases, 68/68). Among the unsuspected test cases, 93.3% (14/15) were correctly identified as AD cases. All Stanford type A cases in the internal test set (*n* = 18) were detected as ADs, and 16 out of 18 detected ADs were additionally flagged as type A. Of the 20 type B cases, 14 were falsely reported as type A. Further inspection revealed that cases in which the originating intimal flap presented in the aortic arch were often classified as type A by the algorithm, and as type B following review by a radiologist. In contrast, automatic Stanford classification during 5-fold cross-validation achieved a sensitivity of 91.5% (54/59) and specificity of 83.3% (5/6) (Table 2, Figs. 4 and 5).

**Table 2:** Performance of the selected model on the different training and test sets

	Internal dataset					External dataset
	Training set	Test set	Atypical cases (test)	Non-suspected cases (test)	Type A cases (test)	Test set
**AUC** **[%] of best-performing model** (95% CI) (label grouping, training with AD-negative cases)	97.02 (94.28–99.76)	98.68 (96.11–100.00)	93.75 (85.38–100.00)	-	–	97.00 (94.66–99.34)
**Sensitivity/specificity** (threshold learned from training set)	95.71 (67/70)/88.51 (77/87)	97.37 (37/38)/100.00 (68/68)	81.25 (13/16)/100.00 (68/68)	93.33 (14/15)/-	100.00 (18/18)/-	92.00 (92/100)/100.00 (38/38)
**Sensitivity/specificity** (human expert 1)	-	92.1% (35/38)/100.0% (68/68)	56.3% (9/16)/100.00 (68/68)	-	66.7% (12/18)/-	-
**Sensitivity/specificity** (human expert 2)	-	89.5% (34/38)/100.0% (68/68)	50.0% (8/16)/100.00 (68/68)	-	72.2% (13/18)/-	-
**Cohen’s Kappa** (threshold learned from training set)	0.83	0.98	0.88	-	-	0.86
**AUPRC** **[%] of best-performing model** (95% CI) (label grouping, training with AD-negative cases)	97.58 (94.91–99.43)	98.87 (96.04–100.00)	91.85 (78.66–100.00)	-	-	99.06 (98.03–99.73)
**Precision/recall** (threshold learned from training set)	94.20 (65/69)/92.86 (65/70)	100.00 (34/34)/89.47 (34/38)	100.00 (13/13)/81.25 (13/16)	86.67 (13/15)/-	94.44 (17/18)/-	100.00 (92/92)/92.00 (92/100)
**Precision/recall** (human expert 1)	-	100.0% (35/35)/92.1% (35/38)	100.0% (9/9)/56.3% (9/16)	-	100.0% (12/12)/66.7% (12/18)	-
**Precision/recall** (human expert 2)	-	100.0% (34/34)/89.5% (34/38)	100.0% (8/8)/50.0% (8/16)	-	92.9% (13/14)/72.2% (13/18)	-
**F1-score** (threshold learned from training set)	93.53	94.44	89.66	-	-	95.83

Sensitivity, specificity, and Cohen’s κ for AD detection are reported along with AUROC, as well as sensitivities in the unsuspected cases subset and on type A test cases only. Additionally, precision and recall for AD detection are reported along with the AUPRC. AUC and AUPRC are reported only for AD *versus* non-AD detection in the underlying datasets. No separate analyses were performed for the unsuspected and type A subtypes within the internal test set. For comparison, the sensitivities, specificities, and precisions achieved by the two human readers are presented for the internal test set, the atypical cases test set, and the type A test cases

*AD* Aortic dissection, *AUROC* Area under the receiver operating characteristic curve, *AUPRC* Area under the precision-recall curve

**Fig. 6:**
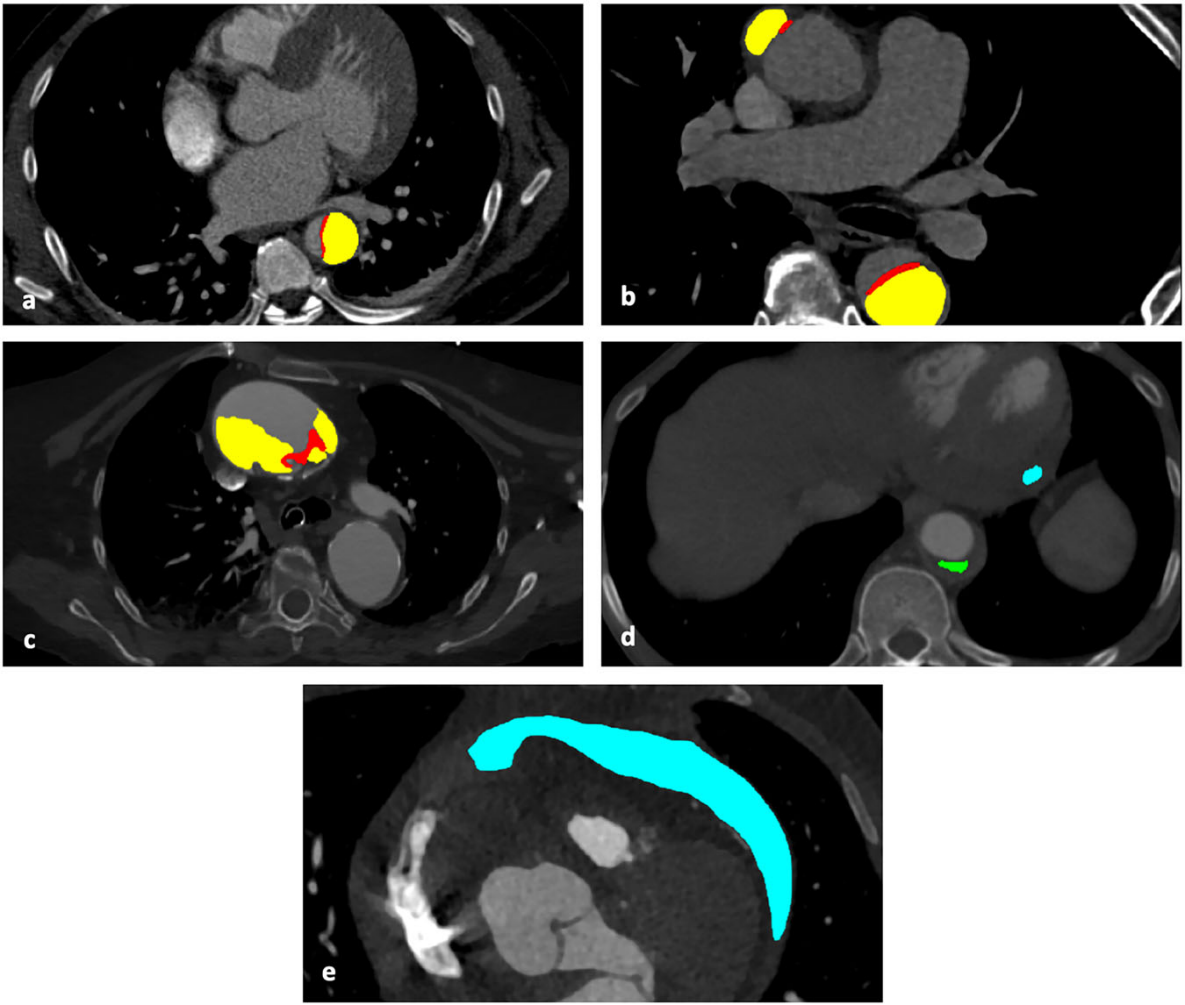
Correctly and incorrectly classified cases. **a** Correctly detected case from the external testing dataset (yellow area: segmentation result of false lumen, red: membrane segmentation). **b** Correct detection of wrongly (AD-negative) labeled AD within the internal test dataset. **c** Correctly detected AD case from the atypical case test dataset, the dissection membrane has collapsed close to the aortic wall and was correctly detected (red), parts of the true lumen were falsely segmented as false lumen (yellow). **d**, **e** Atypical dataset, both false-negative cases not containing dissection membranes, but indirect signs of AD like aortic wall hematoma (green) and hemopericardium (blue). Both cases were later clinically identified as AD cases. AD, Aortic dissection

For AD detection, two human readers achieved sensitivities of 92.1% and 89.5% (34/38) and specificities of 100.0% both. For type A detection, sensitivities were 66.7% (12/18) and 72.2% (13/18), with specificities of 90.0% and 65.0%. In the atypical cases dataset, sensitivities of the two human readers for detecting AD were 56.3% and 50.0%, with precisions of 100.0% both. More details are reported in Table 2. Average computation time was 6:38 min per case (6:29 min for segmentation), depending on input image size. Figure 6 displays cases which AD was identified correctly along with false-negative cases.

The trained network will be publicly available as a non-medical device for further scientific research.

### Discussion

This study demonstrated that, by employing a state-of-the-art deep learning model, reliable automated AD detection on heterogeneous multicenter CT data can be achieved. In this setting, the presented experiments offered valuable insights into detection performance on realistic clinical data containing different contrast phases and complex cases.

Within the clinical context, multiple factors have previously been analyzed with the aim of expediting clinical AD management [14]. The lack of early AD suspicion is a well-known risk factor for delayed AD management. In addition, discrepancies between initial radiological reports and subsequent readings by experts in cardiovascular imaging have been identified as complicating factors. They have been described to occur in 35% of type A and 17% of type B dissections [8]. The deployment of AI-driven techniques holds promise for mitigating these factors and thereby improving the management of acute AD.

Several AI-based tools have been proposed to support emergency management, including automated solutions for treatment stratification, planning, and prognosis [15], triage assistance for chest x-ray reading [16], as well as support for the management of intracranial hemorrhage [17] and pulmonary embolism [18] through analysis of prior CT scans. Beyond these tasks, the automation of AD detection has been pursued with the promise of improving clinical outcomes. Initial efforts focused on automated AD segmentation and included the use of CNNs [19, 20]. Beyond this task, Yi et al [21] proposed a method for automated AD detection, leveraging CNN-derived, patch-level pathology scores in combination with extracted handcrafted morphological features. Their approach achieved accuracy, sensitivity, and specificity of 89.7%, 86.2%, and 92.3% on 341 internal cases and of 73.0%, 97.8%, and 55.4% on 111 external test cases, respectively. Xiong et al [22] proposed a generative modeling approach to perform dissection detection in noncontrast CT data, achieving AUROC, accuracy, sensitivity and specificity of 0.797, 83.1%, 93.8%, and 72.5% on 207 internal cases, and of 0.860, 80.0%, 88.6%, and 72.5% on 30 external cases, respectively. Harris et al [23] proposed a screening algorithm aimed at reducing delays in teleradiology, achieving a sensitivity of 87.8% and a specificity of 90.6% in AD detection. Another method by Hata et al [24] achieved a sensitivity of 91.8% and specificity of 88.2% on selected single-site data from 170 patients, without differentiating between AD subtypes and partly incorporating noncontrast cases. Huang et al [25] developed a two-stage hierarchical neural network on a Taiwanese CT dataset acquired under AD suspicion, which included 51 confirmed AD cases. Their method achieved a sensitivity of 95% and specificity of 99% for type A ADs, though sensitivity dropped to 79% for type B ADs. These results demonstrated that high detection performance and subtype distinction are feasible on single-center data. Yellapragada et al [26] presented a general screening approach for detecting acute aortic pathologies, including dissection, hematoma, and ulcer, by incorporating CNNs and “multiple instance learning”. Trained on 2,291 cases, their method achieved promising AUROCs (0.965 for CNN and 0.985 for multiple instance learning) on 280 single-center cases, which included 50 cases of diseases not exclusive to AD. As for potential limitations, the study focused exclusively on analyzing CTA images and relied on strong assumptions about intra-aortic imaging intensities, which led to relatively strict exclusion criteria for nonprocessable cases. Furthermore, the presented approach performed predictions directly at the patient level, which may complicate the interpretability of results at the pixel level. Laletin et al [27] retrospectively evaluated the AD detection performance of a commercially available automatic solution on a large multi-site dataset comprising 1,303 CTA-only scans, including 137 AD cases, and achieved a sensitivity of 94.2% and a specificity of 97.3%. A recent meta-analysis of studies focusing on the AI-based detection of AD mainly in contrast-enhanced imaging reported a potential best-case sensitivity, specificity, and AUROC of 97%, 93%, and 0.98, reflecting upper bounds of performance reported by the literature [28].

In complement to prior studies, the proposed algorithm shows potential in various regards: methodologically, it specifically leverages strong, state-of-the-art segmentation performance for AD detection, while being remarkably straightforward, relying on U-Net models trained in a fully automated fashion. No strong requirements on input CTs and contrasting were imposed. The proposed aggregation of segmentation labels strengthens robustness against label noise and reader dependency, focusing on the existence of pathological structures during detection rather than their specific delineation. Although promising results have previously been reported on AD detection, its potential on more heterogeneous multicenter data has remained unclear. In this regard, the proposed method achieved similar or better results on a variety of clinical data. Finally, this study provides insights into the robustness of AI results across different scenarios, including cases that were clinically not suspected of AD before imaging, 93.3% of which were detected. Also, all type A dissections were detected, the majority of which were automatically flagged as type A. The comparison with secondary human readers showed that they consistently achieved lower sensitivities on the internal test set, especially on the atypical test set. In many of the missed AD cases, the readers favored alternative diagnoses such as intramural hematoma, suggesting that distinguishing acute aortic pathologies can be challenging under clinical and emergency imaging conditions, where time for interpretation and decision-making is limited. In summary, the presented results indicate a strong potential for the robustness and generalizability of our approach, warranting further validation under real-world conditions.

Widespread clinical implementation of AI has not yet been achieved for several well-documented reasons, including poor integration into existing clinical workflows, insufficient clinical performance, and limited trust in automatic results [29, 30]. Specifically, integrating screening tools inherently requires balancing sensitivity and specificity to align with clinical needs without compromising trust, which calls for careful fine-tuning under clinical conditions. To address the challenge of limited trust in AI-generated results, enhancing the transparency of automated decision-making can be an effective strategy. This may be achieved, for instance, by linking auxiliary algorithmic outputs—such as AD region segmentations [31] or saliency maps highlighting areas relevant to the decision-making process—to the original DICOM data. Seamless and reliable integration of such solutions into AD management workflows may be facilitated by adhering to certified implementation procedures. A key objective could be the targeted deployment of tools for adaptive radiology worklist reprioritization or standalone alert systems, all aligned with existing clinical requirements. This will necessitate seamless technical integration and staff education; otherwise, the potential benefits of using screening tools may be significantly reduced [32]. Finally, for this study, algorithmic developments were aimed at high detection performance rather than fast computation. Further investigation is needed to accelerate predictions while maintaining acceptable detection performance. Several strategies can be explored for this purpose—for example, disabling data augmentation in nnU-Net during inference, which can yield an approximate 8× speedup with minimal loss in segmentation accuracy. Additionally, using state-of-the-art graphics processing unit hardware may provide further speedups in the range of 6×. With these optimizations, application times may be reduced to under 1 min, or even a few seconds per case. Regardless of these options, a consumer-grade graphics processing unit is the minimum requirement for any application of the proposed method in the clinic. This assumption is based on the fact that, for the image sizes encountered in our setting, central processing unit inference times of nnU-Net are expected to be excessively long on a typical desktop workstation. Such high computation times would undermine the practical benefit of automated AD detection in a clinical setting, as proposed in this work. Beyond these runtime considerations for the single case, the proposed method also supports parallel processing of large amounts of imaging data, assuming sufficient hardware resources are available to perform background detection of AD. Altogether, we conclude that the proposed method has potential for enhancing emergency response capabilities in clinical settings.

The results of this study are limited by various factors.

First, limited dataset sizes with comparably high AD-incidence across training and testing datasets may affect generalizability. Also, the study relies on retrospectively collected and curated data, which is prone to selection bias. Algorithm performance was evaluated against human readings conducted under conditions simulating clinical routine. These comparisons do not capture the full spectrum of emergency imaging scenarios encountered in practice. A prospective clinical study would be necessary to accurately evaluate the benefits of deploying the proposed method under extended real-world conditions. In addition, weaker performance was observed in rare cases with atypical anatomy, very short dissection membranes, or imaging reconstructed in lung windowing only. In such cases, patients may be at a disadvantage due to a higher risk of algorithmic misclassification. This highlights that current state-of-the-art methods can reinforce—but not fully replace—human expertise. Maximizing the clinical benefits may thus rely on combining AI-driven automation with expert interpretation. In this context, the presented performance comparison with human readings suggests that the proposed method could be especially valuable for enhancing initial assessments made under emergency imaging conditions. Furthermore, results for atypical and unsuspected AD cases were derived from relatively small sample sizes. This contributes to a greater statistical uncertainty in these results, as reflected by wide confidence intervals. Overall, the presented results were obtained from applying the proposed method to heterogeneous, multicenter data. However, additional research is necessary to evaluate the generalizability of these findings across the broader clinical spectrum. Consequently, a key future objective should be the prospective intra-hospital validation of AI-based detection performance and its clinical impact. Such validation could be performed in a large-scale multicenter setting, encompassing a broader range of individual anatomical variants of AD.

Second, no visual feedback of the algorithm’s reasoning for generating segmentations was integrated, leaving the user with the “black box” problem in case of misdetection. Since any false detection can possibly fuel doubts in AI trustworthiness, integration of features like class activation maps [33] would possibly constitute an important research topic. Third, as acute aortic syndrome consists of additional pathologies like intramural hematoma and aortic ulcer, clinical implementation ultimately requires a broader solution. Finally, refining details such as improving the method’s performance to distinguish between morphologically similar type A and type B dissections could be a focus of future work. Type B cases that were misclassified as Type A by the proposed method presented structurally at the uppermost part of the aortic arch—a pattern underrepresented in the training data, which included only eight Type B cases in total. Including more such cases during training is likely to enhance performance.

In conclusion, we demonstrated that a properly tuned CNN can achieve strong and reliable performance in detecting and segmenting acute AD across heterogeneous, multicenter thoracic CT imaging data. Evaluation on heterogeneous data suggested strong robustness and generalizability, making further testing of potential clinical merits a valuable goal.

## Supplementary information

ELECTRONIC SUPPLEMENTARY MATERIAL

## Data Availability

The datasets used and/or analyzed during the current study are available from the corresponding author on reasonable request.

## Abbreviations

AD: Aortic dissection
AI: Artificial intelligence
AUPRC: Area under the precision-recall curve
AUROC: Area under the receiver operating characteristic curve
CI: Confidence interval
CNN: Convolutional neural network
CT: Computed tomography
CTA: Computed tomography angiography
