# Supplementary material for: Automated AI detection of thoracic aortic dissection on CT imaging
Source: Eur Radiol Exp. 2025 Oct 22;9:102. doi: 10.1186/s41747-025-00640-8 (PMC12546231; doi:10.1186/s41747-025-00640-8)
Supplement: Supplementary file 1 — ELECTRONIC SUPPLEMENTARY MATERIAL [file 41747_2025_640_MOESM1_ESM.pdf]

Automated AI detection of thoracic aortic dissection on CT imaging

ELECTRONIC SUPPLEMENTARY MATERIAL

Index

**Statement on intended purpose / research use**	**1**
**Internal training dataset - patient characteristics and technical details**	**1**
**External testing dataset - details**	**2**
**Additional examples of included internal training cases**	**3**
**Reasoning behind formulating AD detection as semantic segmentation task**	**6**
**Details of the labeling / manual segmentation process**	**6**
**Detailed results for different network training configurations**	**8**
Evaluation and comparison of training configurations	9
**PRIME checklist**	**11**
**CLAIM Checklist**	**13**
**Supplementary sources**	**16**

Statement on intended purpose (research use)

The study was conceptualized as a feasibility study. The trained network will be available for scientific use. The study does not serve as a performance evaluation study for certification of a medical device. The intended use of the algorithm is for research only. It is **not** intended for clinical implementation. The user has to ensure that all local regulations are being met when implementing the software for research purposes.

Internal training dataset - patient characteristics and technical details

**Number of cases**	n=157
**CT slice thickness**	1.57 +- 1.12 (0.3 - 5) mm
**Subjective image quality (1 very good - 5 poor)**	2,21 +- 0,90 (1-5)

Supplementary Table S1: This table displays average slice thicknesses, and Radiologist-rated image quality of the included training cases.

External testing dataset (details)

Supplementary Table S2: Overview of the origins and details of included external test cases.

Public validation dataset		
	AD cases	Non-AD cases
Source	ImageTBAD, Chinese hospital	AVT dataset (described to include cases from the KiTS19 Grand Challenge from Cleveland Clinic, cases from Cancer Imaging Archive originating from Memorial Sloan Kettering Cancer Center and cases from chinese Dongyang Hospital)
Number of cases	n=100	n=38
Average age	52.5 ± 11.3	Unknown
% female	31%	Unknown
CT hardware	Philipps (77%) and Siemens (23%)	Unknown
Slice spacing (mm)	1.0	3.31 +- 1.24 (0.5 - 5)
Size of images	512 x<br>512 x<br>343.8 +- 118.1 (54 - 691) px	512 x<br>580.89 +- 77.60 (512 - 666) x<br>216.68 +- 190.25 (94 - 1059) px
Voxel size	1.0 x 1.0 x 1.0	0.71 +- 0.12 (0.44 - 0.98) x<br>0.71 +- 0.12 (0.44 - 0.98) x<br>3.31 +- 1.24 (0.5 - 5)

Additional examples of included internal training cases

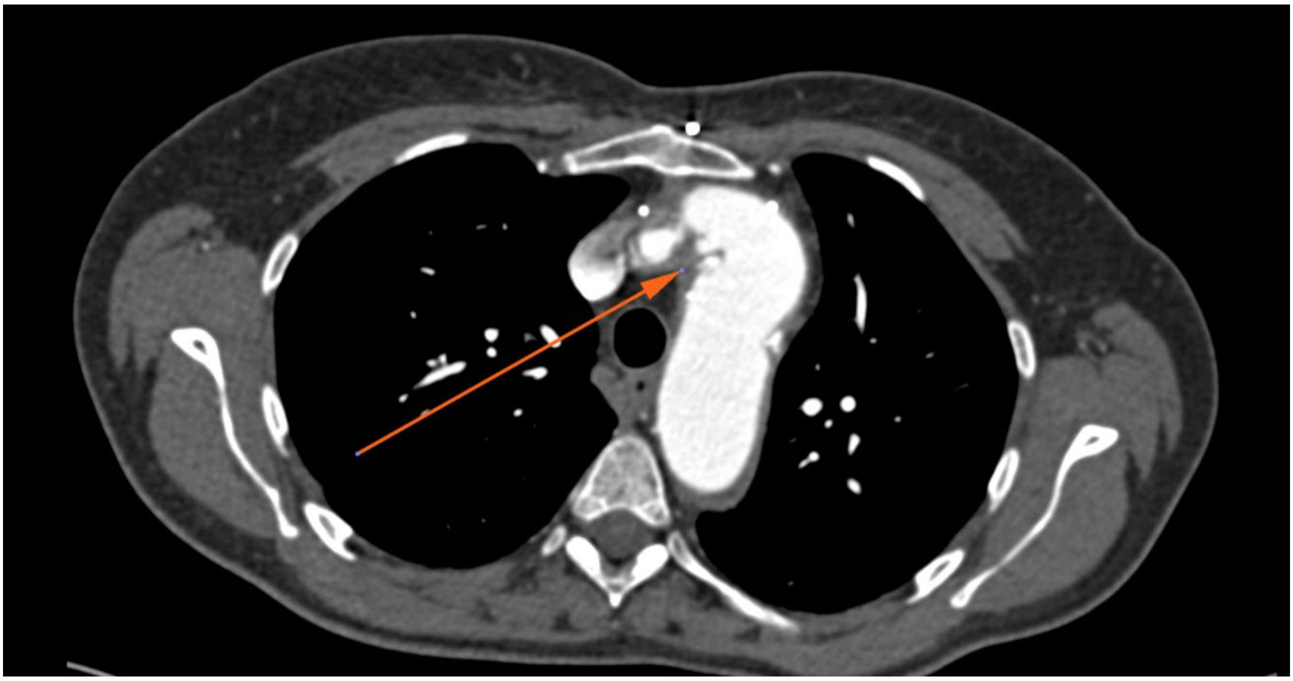

Supplementary Fig. S1: Aortic-wall adherent small dissection membrane (orange arrow).

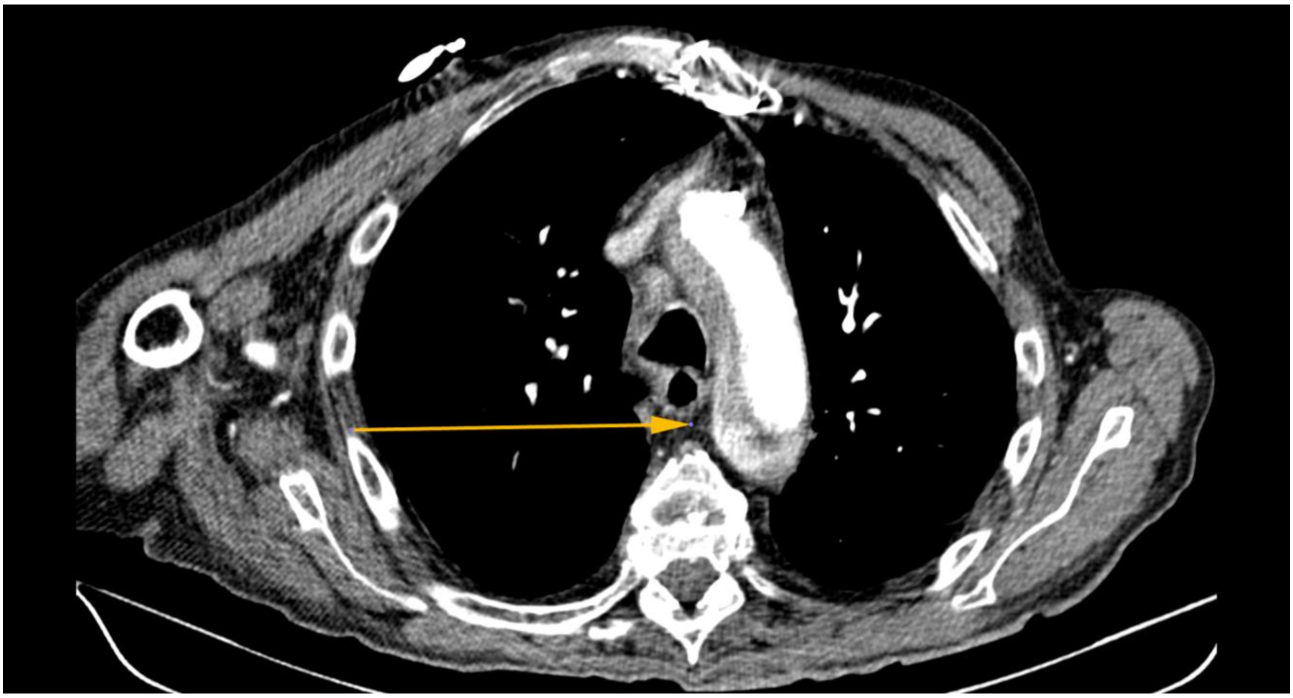

Supplementary Fig. S2: False lumen with only low contrast (yellow arrow) compared to the true lumen.

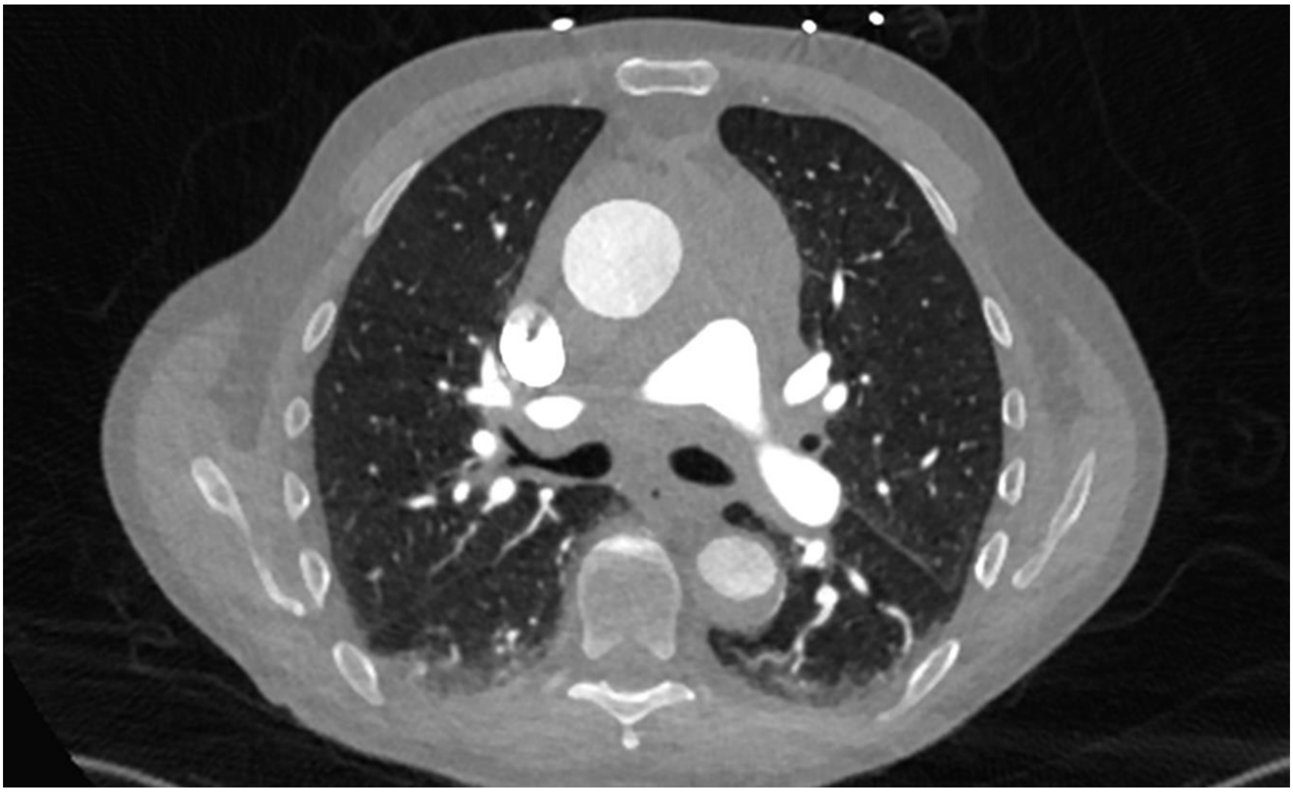

Supplementary Fig. S3: Type B dissection case with presence of aortic wall hematoma.

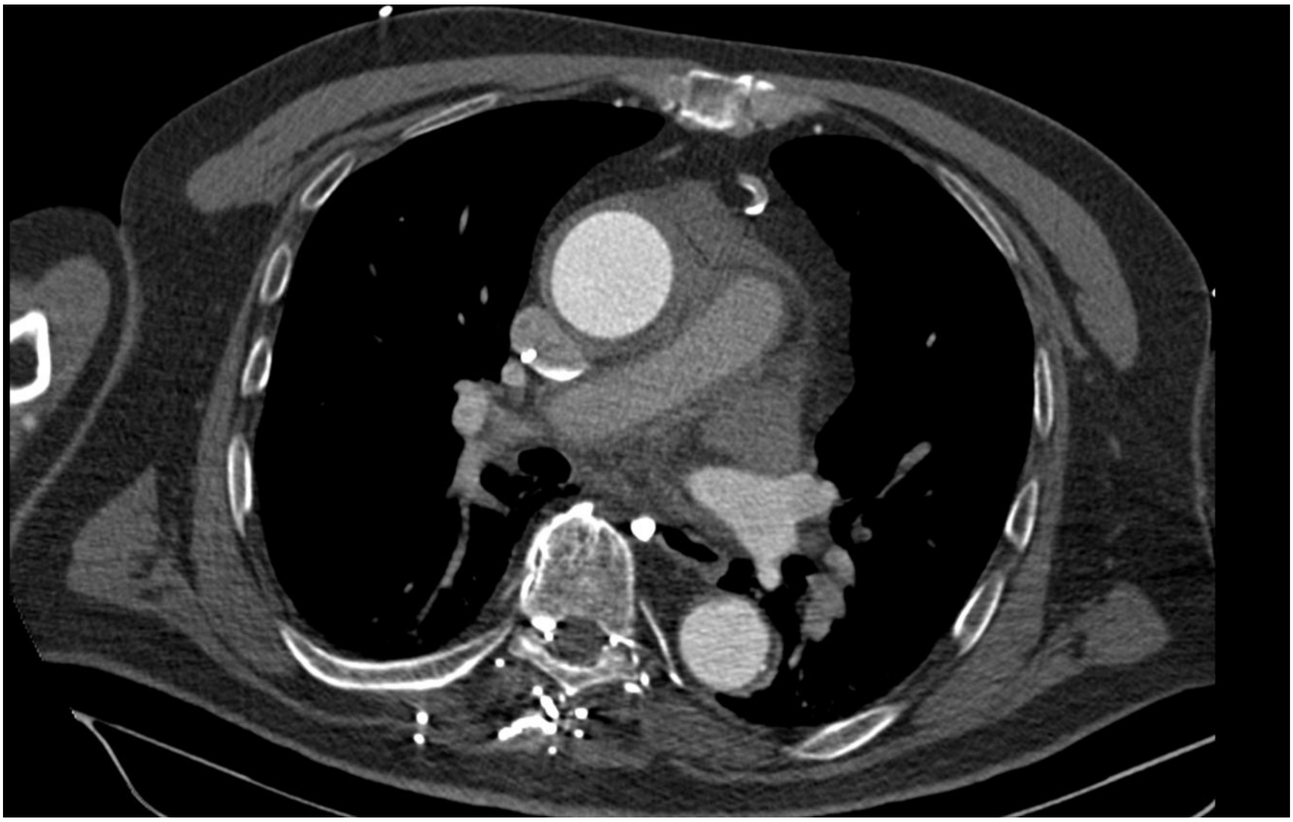

Supplementary Fig. S4: Case with displayed slices not showing the dissection membrane, but only aortic wall hematoma. The more cranial slices contain the dissection.

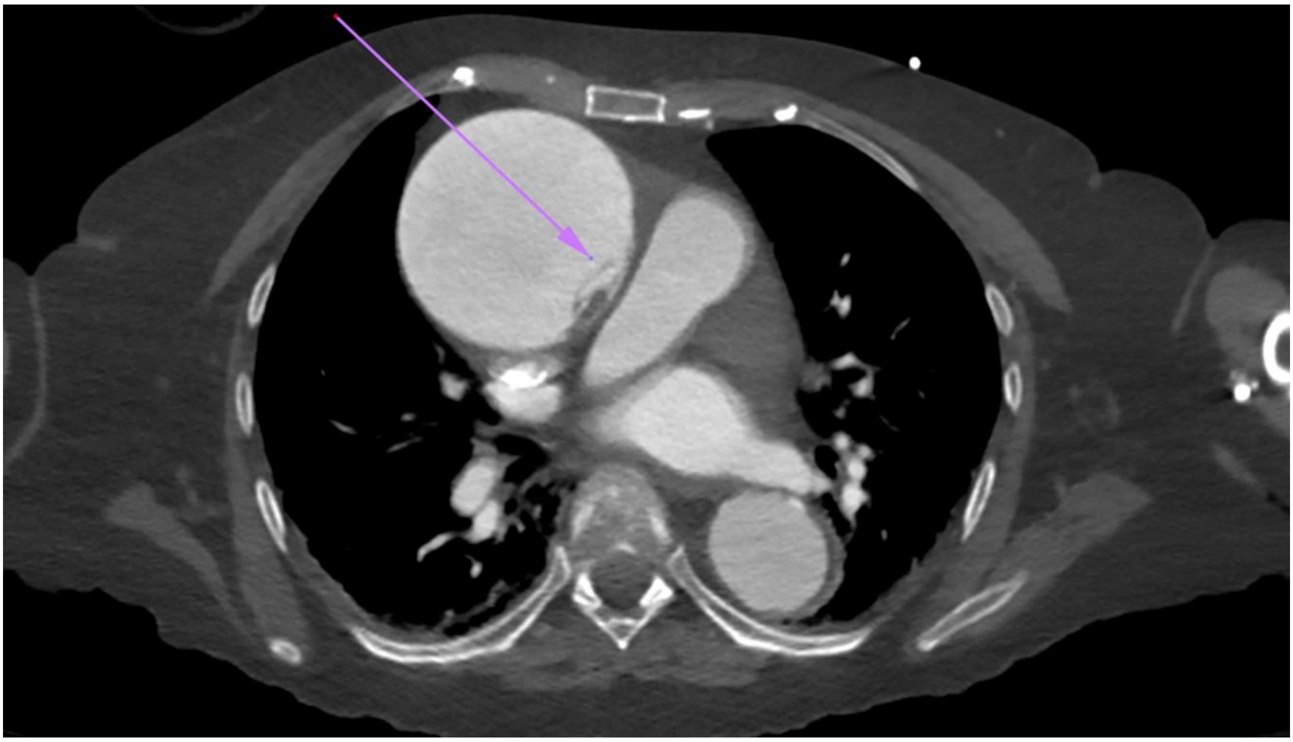

Supplementary Fig. S5. Aortic-wall adherent dissection membrane.

Reasoning behind formulating AD detection as semantic segmentation task

Conceptually, the problem of detecting AD in imaging data can be approached by solving one of several image analysis tasks, including object detection, instance segmentation, semantic segmentation, or image classification. Among these options, object detection and instance segmentation approaches are not ideally suited for performing AD detection: they are primarily designed to locate and annotate multiple, similar objects that can potentially appear in various locations across the entire image. Furthermore, these approaches are most effective at detecting objects that fit evenly into a surrounding bounding box. Both requirements are not met when detecting pathological AD substructures in the thorax, since these typically appear one at a time at two possible locations (i.e. at the ascending and descending part of the aorta), and they comprise elongated and often diagonal serpentine-like structures that cannot compactly be represented by a surrounding bounding box. Image classification approaches on the other hand solely predict presence or absence of AD without explicitly locating or highlighting pathological structures. This however makes their decisions hard to explain, since it is not immediately clear which structures have been found or were overlooked by the system while performing a successful or unsuccessful detection of AD. In contrast to the aforementioned approaches, formulating AD as a semantic segmentation task is more suitable. That is, the goal is defined as a voxel-wise labeling of specific pathological structures that are expected to appear at predetermined image locations, and for which no bounding box representation is needed.

Details of the labeling / manual segmentation process

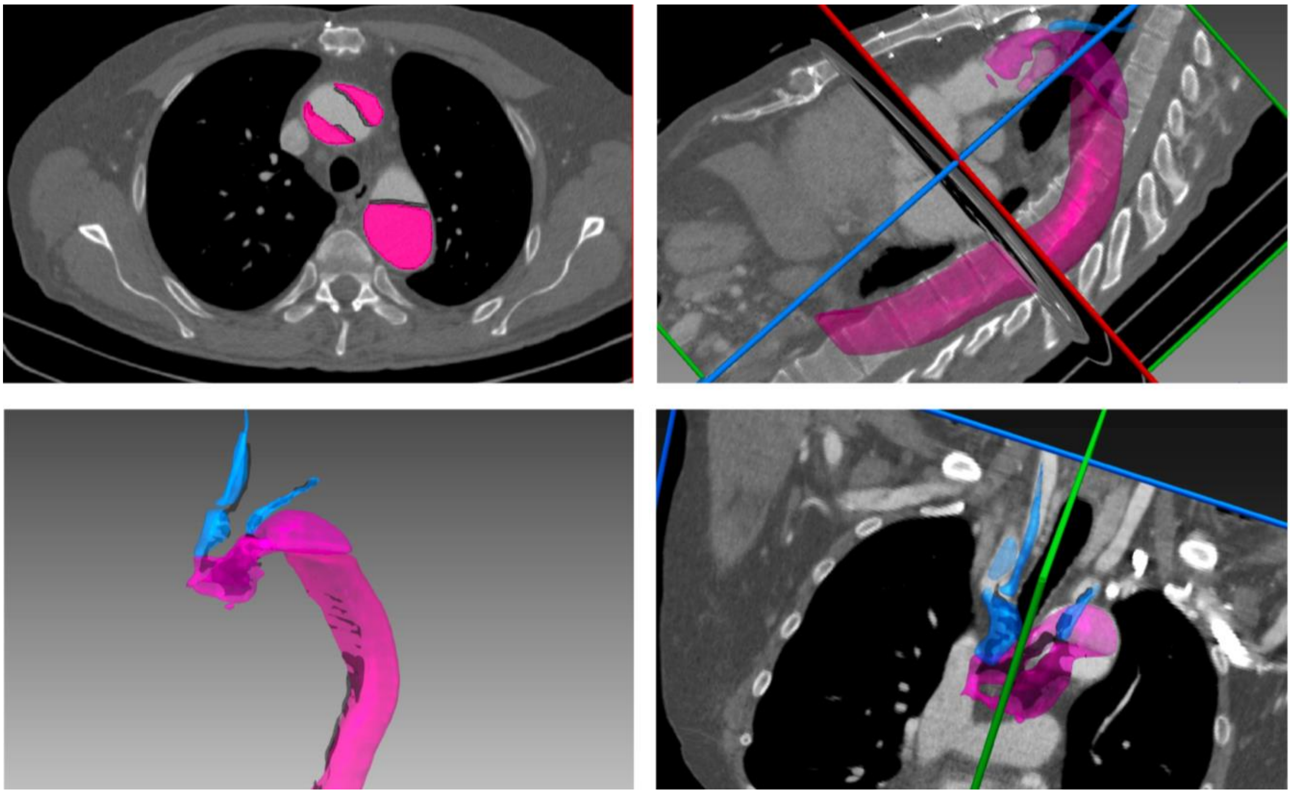

Supplementary Fig. S6: The figure displays the manual training data labeling. According to anatomical presentation of AD cases, labeling was performed on the anonymized CT data. A variety of 10 different labels in total was defined and available, to segment false lumina (ascending / descending), dissection membranes, potential dissection of supra-aortic vessels and indirect signs for AD like hemopericardium or aortic wall hematoma, if present. In cases without AD, no segmentations were performed. The figure exemplarily shows segmentations in one case (strawberry: wrong lumen (different labels for ascending / descending part), black: dissection membrane, blue - segmentation of multiple supra-aortic branches, combined). The bottom left figure shows only the segmentation volumes in a 3D-rendering.

Detailed results for different network training configurations

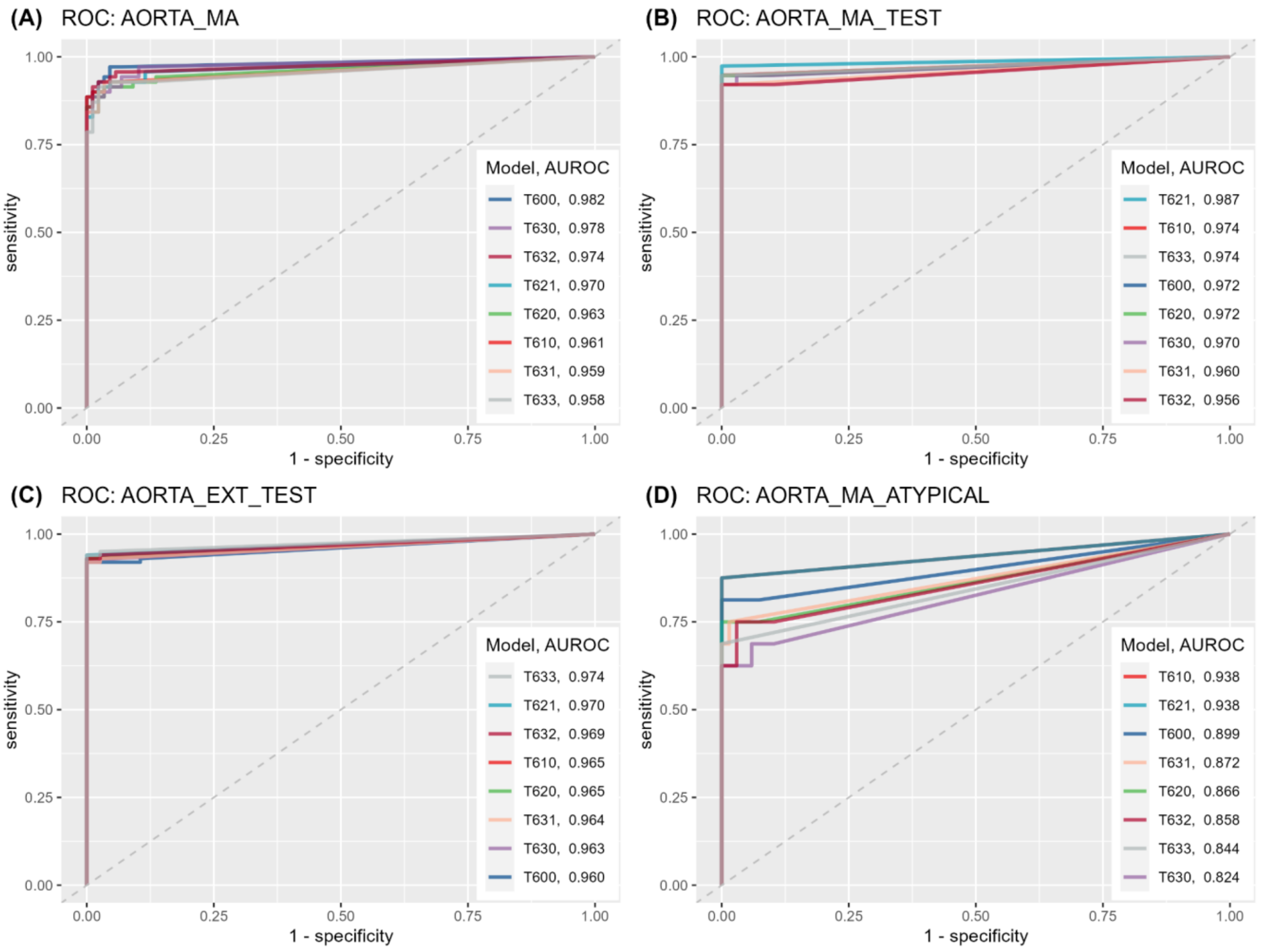

Supplementary Fig. S7: Comparison of algorithm performance across A) Internal training dataset B) Internal test dataset C) external test dataset D) Atypical cases test dataset. The different colors (T600-T633) represent the different training configurations used. T621 (grouped labels, included AD-negative cases) on average yielded best results.

Evaluation and comparison of training configurations

AUROC [%] (95% CI)	MA Internal	MA_independent<br><br>Internal validation set	MA_atypical<br><br>Internal validation set	External validation set	MA-Youden: recall / specificity	Non-suspected cases: recall	MA-stanford (cross validation): recall / specificity
all_labels	98.21 (96.09-100.00)	97.17 (93.31-100.00)	89.94 (79.32-100.00)	96.03 (93.21-98.84)	94.74 (36/38) / 94.12 (64/68)	93.33 (14/15)	80.33 (49/61) / 85.71 (6/7)
all_labels_healthy	96.09 (92.87-99.31)	97.37 (93.77-100.00)	93.75 (85.38-100.00)	96.50 (93.99-99.01)	89.47 (34/38) / 100.00 (68/68)	86.67 (13/15)	91.38 (53/58) / 80.00 (4/5)
label_groups	96.26 (93.08-99.45)	97.17 (93.31-100.00)	86.58 (74.79-98.37	96.50 (93.99-99.01)	94.74 (36/38) / 100.00 (68/68)	93.33 (14/15)	94.92 (56/59) / 80.00 (4/5)
label_groups_healthy	97.02 (94.28-99.76)	98.68 (96.11-100.00)	93.75 (85.38-100.00)	97.00 (94.66-99.34)	97.37 (37/38) / 100.00 (68/68)	93.33 (14/15)	91.53 (54/59) / 83.33 (5/6)
3_labels	97.76 (95.48-100.00)	97.02 (93.05-100.00)	82.40 (69.49-95.31)	96.29 (93.63-98.95)	92.11 (35/38) / 98.53 (67/68)	86.67 (13/15)	93.10 (54/58) / 75.00 (3/4)
3_labels_healthy	95.91 (92.61-99.21)	95.99 (91.58-100.00)	87.22 (76.12-98.33)	96.38 (93.79-98.97)	89.47 (34/38) / 100.00 (68/68)	86.67 (13/15)	93.22 (55/59) / 83.33 (5/6)
3_labels_membrane_first	97.41 (94.79-100.00)	95.65 (90.85-100.00)	85.85 (73.79-97.90)	96.89 (94.49-99.30)	92.11 (35/38) / 100.00 (68/68)	86.67 (13/15)	96.61 (57/59) / 83.33 (5/6)
3_labels_membrane_first_healthy	95.76 (92.34-99.17)	97.37 (93.77-100.00)	84.38 (72.65-96.10)	97.41 (95.20-99.62)	89.47 (34/38) / 100.00 (68/68)	86.67 (13/15)	91.53 (54/59) / 80.00 (4/5)

Supplementary Table S3: This table shows the results (AUROC in %, 95% Confidence Interval according to DeLong's test) of the different network training approaches (rows) on the different datasets (columns). The approach with grouped labels and inclusion of non-AD cases ("label_groups_healthy", yellow background) showed best general performance and was therefore selected for further evaluation.

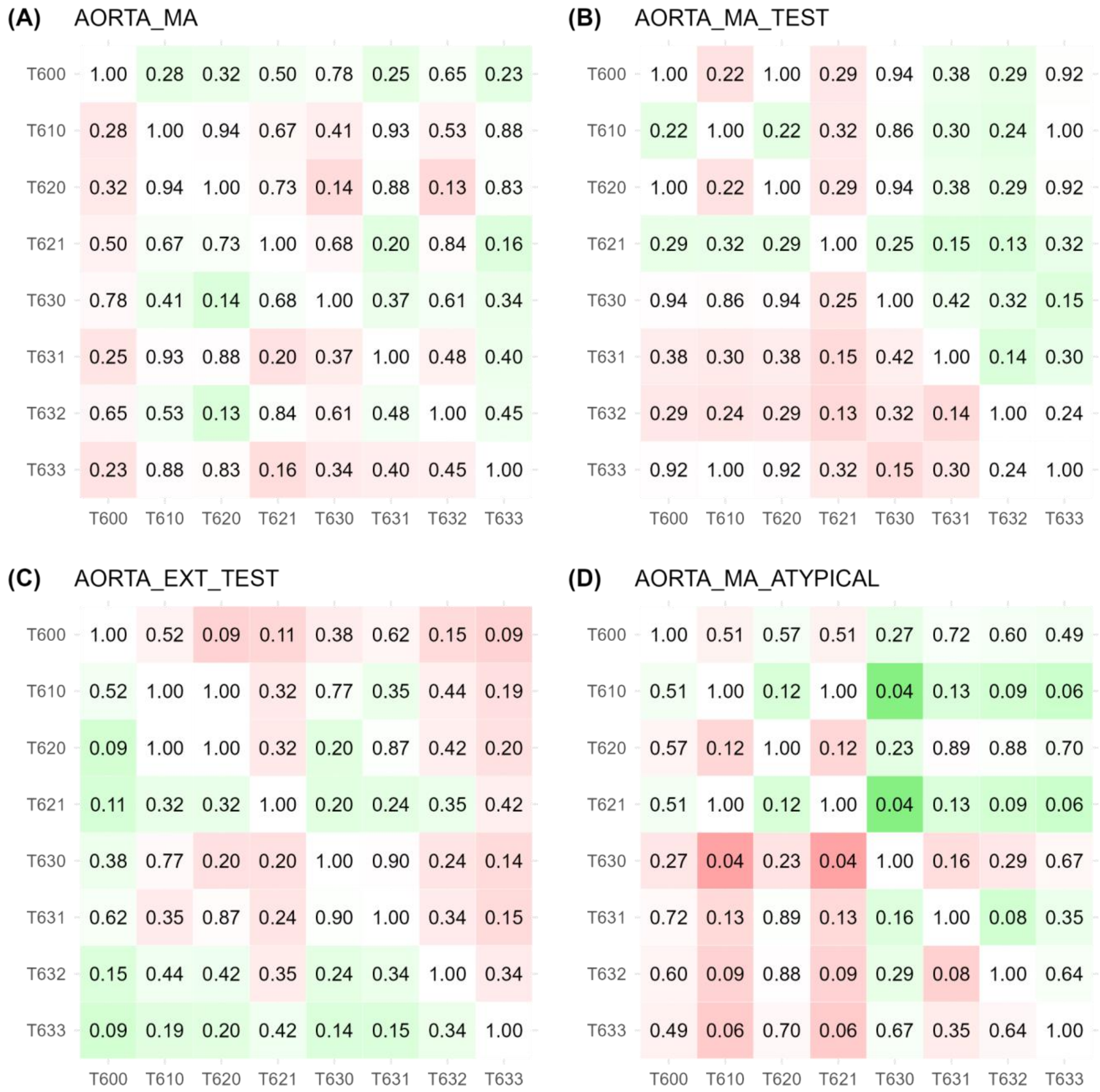

Supplemental Fig. 8: DeLong's test p-values for AUC comparisons across all model versions and datasets. Only on the test dataset of atypical cases, statistically significant differences ($p<0.05$) were observed, which support the selection of the chosen model configuration (T621). No p-value correction (e.g., Bonferroni method) was applied, as the p-values were used to indicate model preference, and the universal null hypothesis was not applicable in this context.

Supplementary Table S4: Adherence to internationally accepted reporting standards for the machine learning model was documented by the PRIME checklist [1]

Section	Checklist item	This study<br>Chapter / paragraph p x
1	Designing the study plan	
1.1	Describe the need for the application of machine learning to the dataset	Introduction / p 1-3
1.2	Describe the objectives of the machine learning analysis	Introduction / p 4
1.3	Define the study plan	Methods - all parts
1.4	Describe the summary statistics of baseline data	Results - / p 1
1.5	Describe the overall steps of machine learning workflow	Methods / p 6,10
2	Data standardization, feature engineering, and learning	
2.1	Describe how the data were processed in order to make it clean, uniform, and consistent	Methods / p 3-4
2.2	Describe whether variables were normalized and if so, how this was done	Methods, p 6-7 (Neural network training)
2.3	Provide details on the fraction of missing values (if any) and imputation methods	Results / p 1;<br>Supplements / Fig. 1
2.4	Perform and describe feature selection process	Methods / p 6 (Neural network training)
2.5	Identify and describe the process to handle outliers if any	N/A
2.6	Describe whether class imbalance existed, and which method was applied to deal with it	Methods / p 9
3	Selection of Machine Learning Model	
3.1	Explicitly define the goal of the analysis e.g., regression, classification, clustering	Abstract / Objectives;<br>Introduction / p 3,4
3.2	Identify the proper learning method used (e.g., supervised, reinforcement learning etc.) to address the problem	Methods / p 1
3.3	Provide explicit details on the use of simpler, complex, or ensemble models	Methods / p 6 (Neural network training)
3.4	Provide the comparison of complex models against simpler models if possible	Discussion / p 3,4
3.5	Define ensemble methods, if used	Methods / p 6 (Neural network training)
3.6	Provide details on whether the model is interpretable	Discussion - p 6 (Limitations)
4	Model Assessment	
4.1	Provide a clear description of data used for training, validation, and testing	Results / p 1;<br>Table 1; Methods / p 6 (Neural network training)

4.2	Describe how the model parameters were optimized (e.g., optimization technique, number of model parameters etc.)	Methods / p 6 (Neural network training)
5	Model Evaluation	
5.1	Provide the metric(s) used to evaluate the performance of the model	Methods / p 12 (Algorithm performance measurement)
5.2	Define the prevalence of disease and the choice of the scoring rule used	Introduction / p1<br>Methods / p 8 (Classifier decision making)
5.3	Report any methods used to balance the numbers of subjects in each class	Methods / p 3
5.4	Discuss the risk associated to misclassification	Discussion / p 6 (Limitations)
6	Best Practices for Model Replicability	
6.1	Consider sharing code or scripts on public repository with appropriate copyright protection steps for further development and non-commercial use	Done
6.2	Release data dictionary with appropriate explanation of the variables	N/A (patient data sharing is restricted)
6.3	Document version of all software and external libraries	Done, within manuscript.
7	Reporting limitations, biases and alternatives	
7.1	Identify and report the relevant model assumptions and findings	Discussion
7.2	If well performing models were tested on a hold-out validation dataset, detail the data of that validation set with the same rigor as that of training dataset (see section 2 above)	Table 1

Supplementary Table S5: Adherence to internationally accepted reporting standards for the machine learning model was documented by the CLAIM checklist [2]

Section / Topic	No.	Item	Section / p = paragraph number
TITLE / ABSTRACT			
	1	Identification as a study of AI methodology, specifying the category of technology used (e.g., deep learning)	Abstract - Methods
	2	Structured summary of study design, methods, results, and conclusions	Abstract
INTRODUCTION			
	3	Scientific and clinical background, including the intended use and clinical role of the AI approach	Introduction / p 1-4
	4	Study objectives and hypotheses	Introduction / p4
METHODS			
Study Design	5	Prospective or retrospective study	Methods / p2
	6	Study goal, such as model creation, exploratory study, feasibility study, non-inferiority trial	Methods / p1
Data	7	Data sources	Methods / p3,4
	8	Eligibility criteria: how, where, and when potentially eligible participants or studies were identified (e.g., symptoms, results from previous tests, inclusion in registry, patient-care setting, location, dates)	Methods / p3
	9	Data pre-processing steps	Methods / p5
	10	Selection of data subsets, if applicable	Methods / p4
	11	Definitions of data elements, with references to Common Data Elements	Methods / p3
	12	De-identification methods	Methods /p3
	13	How missing data were handled	Methods / p3, Supplemental Fig. 1
Ground Truth	14	Definition of ground truth reference standard, in sufficient detail to allow replication	Methods / p3,5
	15	Rationale for choosing the reference standard (if alternatives exist)	N/A
	16	Source of ground-truth annotations; qualifications and preparation of annotators	Methods / p5
	17	Annotation tools	Methods / p5
	18	Measurement of inter- and intrarater variability; methods to mitigate variability and/or resolve discrepancies	Methods /p5
Data Partitions	19	Intended sample size and how it was determined	Methods / p1
	20	How data were assigned to partitions; specify proportions	Methods / p4
	21	Level at which partitions are disjoint (e.g., image, study, patient, institution)	Methods / p4, Table 1
Model	22	Detailed description of model, including inputs, outputs, all intermediate layers and connections	Methods / p6,7
	23	Software libraries, frameworks, and packages	Methods / p6

	24	Initialization of model parameters (e.g., randomization, transfer learning)	Methods / p6
Training	25	Details of training approach, including data augmentation, hyperparameters, number of models trained	Methods / p8
	26	Method of selecting the final model	Methods / p12, Results / p3
	27	Ensembling techniques, if applicable	Methods / p6
Evaluation	28	Metrics of model performance	Methods / p12
	29	Statistical measures of significance and uncertainty (e.g., confidence intervals)	N/A
	30	Robustness or sensitivity analysis	Methods / p12
	31	Methods for explainability or interpretability (e.g., saliency maps), and how they were validated	N/A
	32	Validation or testing on external data	Methods / p4
RESULTS			
Data	33	Flow of participants or cases, using a diagram to indicate inclusion and exclusion	Supplemental Fig. 1
	34	Demographic and clinical characteristics of cases in each partition	Table 1, Results / p1
Model performance	35	Performance metrics for optimal model(s) on all data partitions	Results / p3
	36	Estimates of diagnostic accuracy and their precision (such as 95% confidence intervals)	N/A
	37	Failure analysis of incorrectly classified cases	Results / p4
DISCUSSION			
	38	Study limitations, including potential bias, statistical uncertainty, and generalizability	Discussion / p6
	39	Implications for practice, including the intended use and/or clinical role	Discussion / p4
OTHER INFORMATION			
	40	Registration number and name of registry	N/A
	41	Where the full study protocol can be accessed	N/A
	42	Sources of funding and other support; role of funders	Funding declared

Supplementary references

1. Sengupta PP, Shrestha S, Berthon B, et al (2020) Proposed Requirements for Cardiovascular Imaging-Related Machine Learning Evaluation (PRIME): A Checklist. JACC Cardiovasc Imaging 13:2017–2035. https://doi.org/10.1016/j.jcmg.2020.07.015
2. Mongan J, Moy L, Kahn CE (2020) Checklist for Artificial Intelligence in Medical Imaging (CLAIM): A Guide for Authors and Reviewers. Radiol Artif Intell 2:e200029. https://doi.org/10.1148/ryai.2020200029
